# Harnessing the biological complexity of Big Data from LINCS gene expression signatures

**DOI:** 10.1371/journal.pone.0201937

**Published:** 2018-08-29

**Authors:** Aliyu Musa, Shailesh Tripathi, Meenakshisundaram Kandhavelu, Matthias Dehmer, Frank Emmert-Streib

**Affiliations:** 1 Predictive Medicine and Data Analytics Lab, Department of Signal Processing, Tampere University of Technology, Tampere, Finland; 2 Molecular Signaling Lab, Faculty of Biomedical Sciences and Engineering, Tampere University of Technology, Tampere, Finland; 3 University of Applied Sciences Upper Austria, Steyr, Austria; 4 BioMediTech Institute, Tampere University of Technology, Tampere, Finland; 5 Institute for Bioinformatics and Translational Research, UMIT- The Health and Life Sciences University, Hall in Tyrol, Austria; Institute of Molecular and Cell Biology, SINGAPORE

## Abstract

Gene expression profiling using transcriptional drug perturbations are useful for many biomedical discovery studies including drug repurposing and elucidation of drug mechanisms (MoA) and many other pharmacogenomic applications. However, limited data availability across cell types has severely hindered our capacity to progress in these areas. To fill this gap, recently, the LINCS program generated almost 1.3 million profiles for over 40,000 drug and genetic perturbations for over 70 different human cell types, including meta information about the experimental conditions and cell lines. Unfortunately, Big Data like the ones generated from the ongoing LINCS program do not enable easy insights from the data but possess considerable challenges toward their analysis. In this paper, we address some of these challenges. Specifically, first, we study the gene expression signature profiles from all cell lines and their perturbagents in order to obtain insights in the distributional characteristics of available conditions. Second, we investigate the differential expression of genes for all cell lines obtaining an understanding of condition dependent differential expression manifesting the biological complexity of perturbagents. As a result, our analysis helps the experimental design of follow-up studies, e.g., by selecting appropriate cell lines.

## Introduction

Despite continuous progress in our understanding of the genetic origin of diseases our ability of treating and curing such diseases lacks far behind [1–5]. For this reason, it has been proposed to utilize genomic information for the development of drugs to directly translate results from basic research to clinical applications [6, 7]. A particular example of such a genome-scale project is the Library of Integrated Network-based Cellular Signatures (LINCS) program [8].

The LINCS program [8] (https://clue.io), generated genetic and molecular signatures of human cell lines in response to a variety of perturbations. Specifically, a vast library of gene expression profiles that includes over one million experiments covering more than seventy human cell lines has been generated by measuring the expression values for 978 landmark genes, hence, called the LINCS L1000 data. These data include experiments using over 20,000 chemical perturbagens (small drug molecules), namely drug compounds added to the cell culture to induce changes in the gene expression profile. In addition, there are genetic perturbation experiments targeting a single gene to control its expression level, either suppressing it (knockdown) or enhancing it (overexpression). The LINCS L1000 data is publicly available for download from (https://clue.io/data) and from the Gene Expression Omnibus (GEO) database with accession number GSE92742 (https://www.ncbi.nlm.nih.gov/geo/query/acc.cgi?acc=GSE92742).

The LINCS L1000 data provide an unprecedented compendium of both structural and transcriptomic drug data. However, the availability of such *Big Data* [9, 10] like LINCS L1000, provide also major challenges for their analysis requiring the development of novel approaches and methods. Examples of such approaches for exploring the LINCS L1000 data can be found in [11]. This study focused on finding structural similarities of drugs with a combination of 3D molecular structure to show significant associations of drugs with similar transcriptional changes, supporting the usage of drug-related data [11]. Another study showed that perturbational data can be used for finding common and cell-type specific responses to anti-cancer drug [12]. One major challenge in drug discovery is identifying biochemical interactions of small drug molecules [13]. For this reason, vast effort has been put into discovering the drug MoA and understanding the genetic interactions within cells that will lead to a much fuller understanding of how organisms develop interactions at a cellular level, as well as how diseases such as cancer affect cells and how they can be treated [14, 15]. Several methods such as high-throughput screen is used in identifying interactions of small drug molecules showing activity in biological assays (cellular assays, enzyme activity assays, binding assay) for a single therapeutic target or pathway of interest [16–18]. These examples show the vast use of such data in drug discovery applications.

One problem of the LINCS program is that it constitutes an ongoing endeavor. That means at present there is no foreseeable end when the last samples are deposited. This feature is shared with other genomic data repositories, e.g., Gene Expression Omnibus (GEO) [19], Protein Data Bank (PDB) [20] or Reactome (database of reactions, pathways and biological processes) [21]. All of these data repositories have in common that the data have not been generated from one laboratory sponsored by one funding agency, but multiple independently funded laboratories generated and are still generating data to date. As a consequence, the information contained in such repositories and also in LINCS is a function of time. A problem resulting from this and the fact that multiple laboratories contribute to these data is the lack of global overview statistics that characterize the content of the data. This lack of overview statistics hampers the downstream usage of the LINCS L1000 data for any data analytics application, as outlined above, severely because essentially any statistical data analysis requires knowledge of available sample sizes and available experimental conditions in order to design an analysis properly [22, 23]. For instance, one would like to know how many experiments have three or more replicates for cell line HA1E? Or how many samples are available for cell line A375 having been exposed to four different drug dosages? These and similar questions are currently unanswered and there is no simple way for obtaining such information. For this reason regular updates of the content of such data repositories need to be provided in order to inform the community.

In this paper, we address this problem by exploring and summarizing the LINCS L1000 data as provided by the signature profiles. Specifically, we analyze the LINCS L1000 data for two different layers. In the first layer we focus on the signature profiles themselves and in the second layer we focus on the differentially expression of genes derived from the signature profiles. This means we are moving from overview distributions on a basic level to characterizations of the biological activity of the cell lines in dependence on multivariate conditions, as given by, e.g., the number of replicates or the duration of applied drug perturbations. This will allow to gain insights into the distributions of cell types, time points and small drug molecule dosages across multiple compounds and all experiments conducted so far.

## Methods

### LINCS L1000 dataset

The LINCS L1000 dataset comprises 5806 genetic perturbations (e.g., single gene knockdown and over-expression) and 16,425 perturbations induced by chemical compounds (e.g., drugs) [24]. So far about 1.3 million gene expression profiles have been generated and collected for this project using the L1000 technology [25]. The L1000 platform has been developed at the Broad Institute by the connectivity map (CMap) team to facilitate rapid, flexible and high-throughput gene expression profiling at lower costs. Specifically, this means the L1000 technology measures expression for 978 *landmark* genes and expression values for the remaining transcriptome is estimated using a computational model based on data from the Gene Expression Omnibus (GEO) [26].

### Metadata pipeline

The LINCS data API provides a programmatic pipeline to annotations and perturbational signatures in the L1000 dataset via a collection of HTTP-based RESTful web services. An example for such a service is ‘Cell Service’, which is a service that describes the cell line meta-information. Table 1 lists all the API services provided by the LINCS API for querying the L1000 metadata. These services support complex queries via simple HTTP GET requests that can be executed in a web browser or with most programming languages.

**Table 1:** List of LINCS L1000 metadata APIs.

Service	Description	URL link
Cell Service	The Cell information service returns cell line information.	https://clue.io/api#cells
Gene Service	The Gene information service returns meta-information for measured and inferred genes in the LINCS dataset.	https://clue.io/api#genes
Profile Service	The Profile information service returns meta-information for instances in the LINCS dataset.	https://clue.io/api#profiles
Pert Service	The Pert information service returns meta-information for perturbations in the LINCS dataset.	https://clue.io/api#perts
Plate service	The PlateInfo service returns plate information.	https://clue.io/api#plates
Signatures	The Signature information service returns meta-information for signatures in the LINCS dataset.	https://clue.io/api#signatures

## Results

The LINCS L1000 data is a vast collection of gene expression profiles and meta information that includes many experimental samples covering more than seventy human cell lines. These cell lines are populations of cells descended from an original source cell and having the same genetic make-up, kept alive by growing them in a culture separate from their original source [27]. In the following, we analyze the LINCS L1000 data for two different layers. The first layer focuses on the signature profiles themselves and the second layer on the differentially expression of genes derived from the signature profiles. This means we are moving from overview distributions on a basic level to characterizations of the biological activity of the cell lines in dependence on multivariate conditions, as given by, e.g., the number of replicates or the duration of applied drug perturbations. Hence, this provides an understanding of the biological functions effected by the perturbations.

### A. Signature profiles

#### Cell line and small molecule annotations

**Fig 1:**
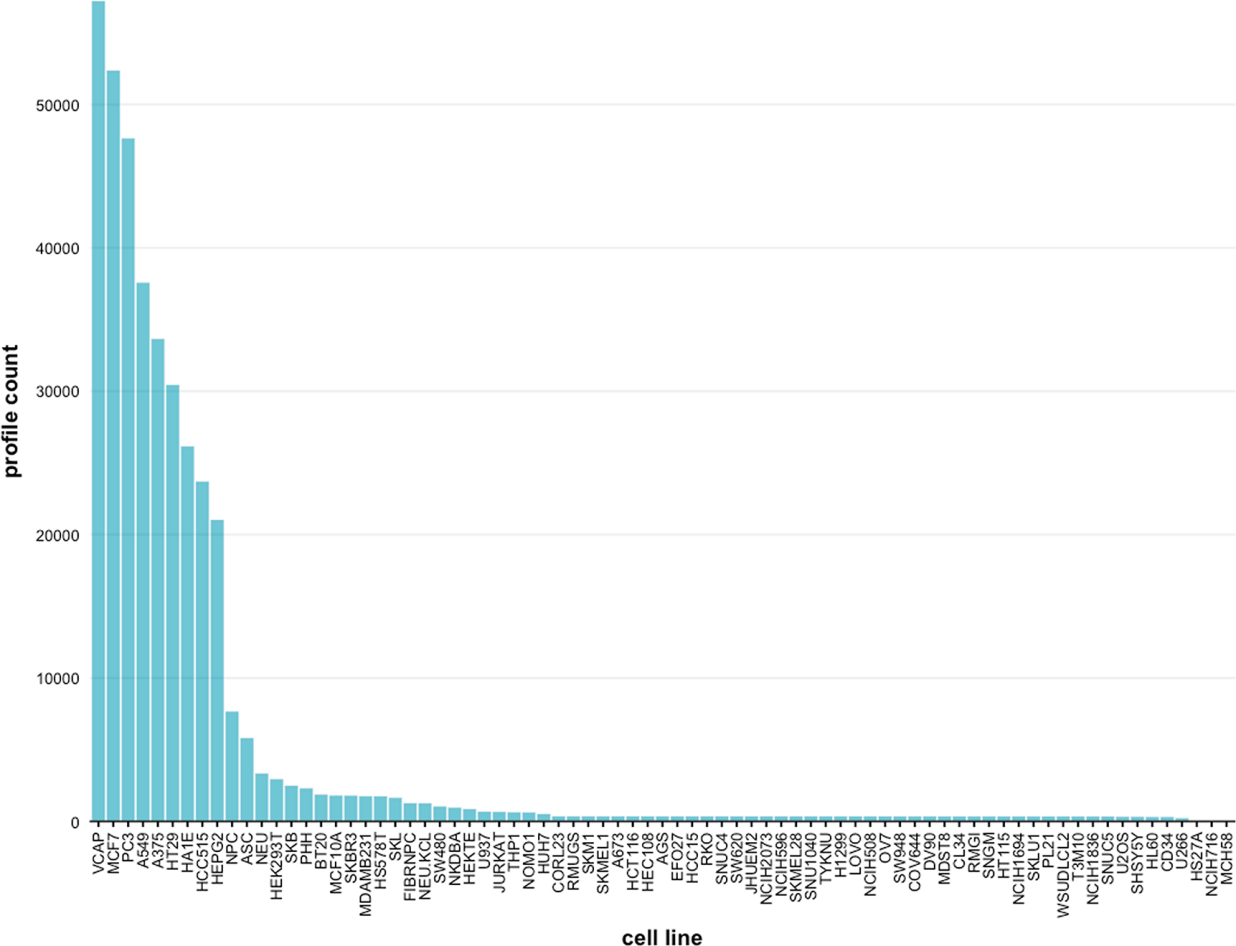
Cell line signature profile counts. The drug signature profile count distribution is shown for all 71 cell lines across all experiments in the LINCS L1000 dataset. Each bar gives the number of available signature profiles per cell line.

Various cancer cell lines and non-transformed primary cultures were used to represent disease models in the LINCS L1000 data [28]. To enable an integration and analysis of large cell-based screening profiles in the LINCS project, the cell lines were annotated with labeled terms to identify the associated organs and diseases. In Fig 1 we show the overall distribution of profiled samples for 71 cell lines across all experiments. These counts include all the corresponding cell line profiles. For obtaining this information, we used the metadata annotations that are available via the Cell Service API. By summation over all cell lines in Fig 1 we find that, currently, the total number of signature profiles (excluding the profiles treated with knockdown and overexpression genes) is 215,224. This number is much smaller than the 1.3 million raw gene expression samples because the replicated raw sample have been summarized for obtaining the signature profiles resulting from a comparison of treatment with control conditions.

From Fig 1 it is clear to see that there are many cell lines that are not highly profiled and therefore have low profile counts. For this reason, in the following we focus on the 9 cell lines with the highest profile counts. In Table 2 we show the count distribution of these 9 cell lines, each containing more than 20,000 profiles.

**Table 2:** Cell lines with the highest number of available signature profiles in the LINCS L1000 data and their corresponding annotation according to the Cell Service API.

Cell line	Profile count	Tissue
A375	33,656	Skin
A549	37,577	Lung
HCC515	23,714	Lung
HA1E	26,164	Kidney
HEPG2	21,032	Liver
HT29	30,449	Colon
MCF7	52,373	Breast
PC3	21,032	Prostate
VCAP	21,032	Prostate

**Fig 2:**
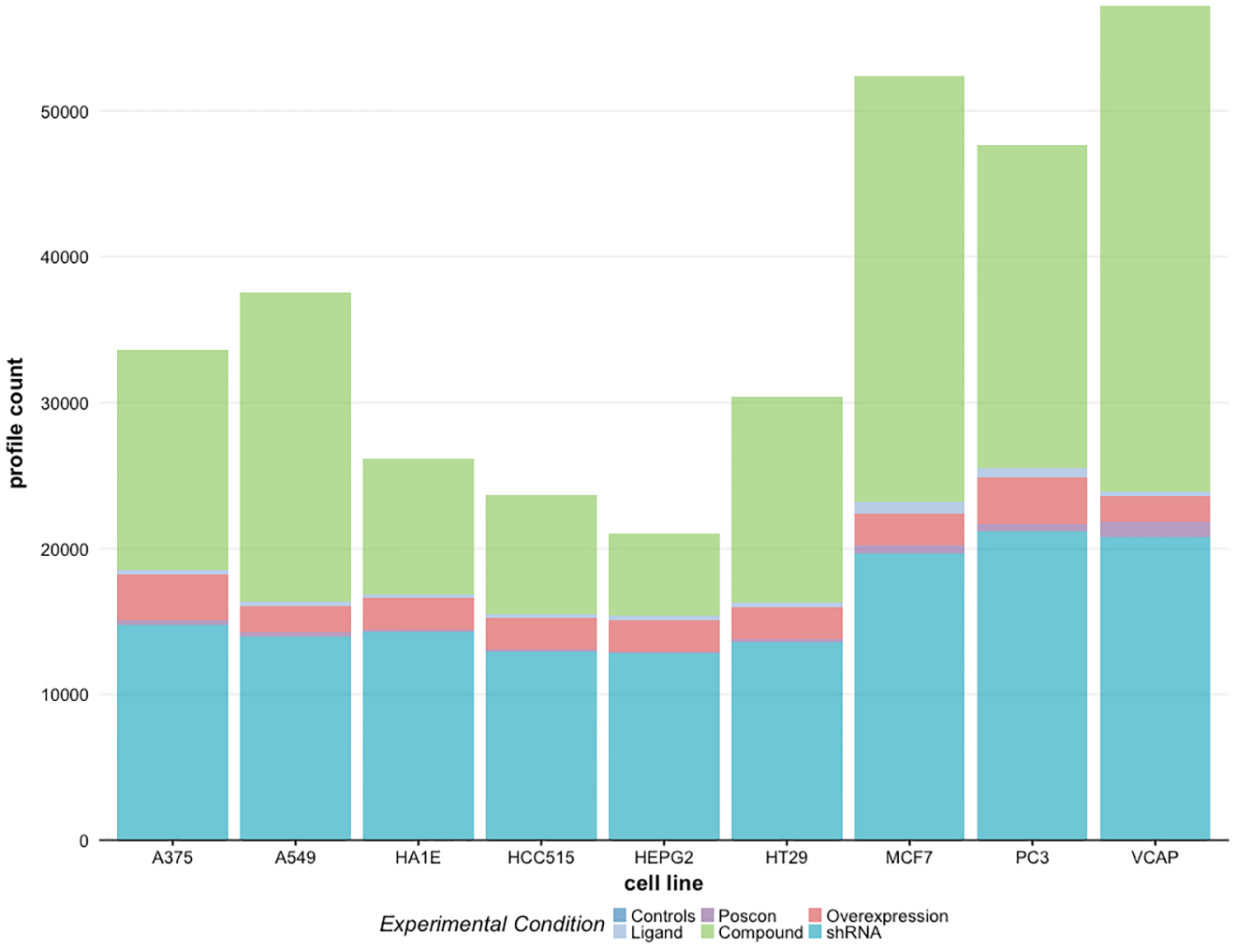
Distribution of experimental conditions for 9 highly profiled cell lines. Each stack bar shows the proportion of available profiles for different small molecules and controls used for the experimental condition.

The LINCS L1000 data include experiments for more than 20,000 small molecule perturbations. The perturbations are applied to the cell culture to induce changes in the gene expression profiles. Furthermore, there are genetic perturbation experiments targeting single genes to control their expression levels, by either suppressing or enhancing them [29]. Detailed information for small molecule perturbations can be retrieved using the Pert Service API that identifies unique and common drugs used in the L1000 dataset. In Fig 2 we show the count distribution of 6 different treatment and control samples including genetic and small molecule perturbations. The count distributions shown correspond to the same 9 cell lines as in Table 2. The 6 experimental conditions considered are: controls, ligands, poscons, compounds, overexpression and shRNAs. As one can see the number of controls and compounds is always highest for all cell lines followed by the number of overexpressed profiles.

**Fig 3:**
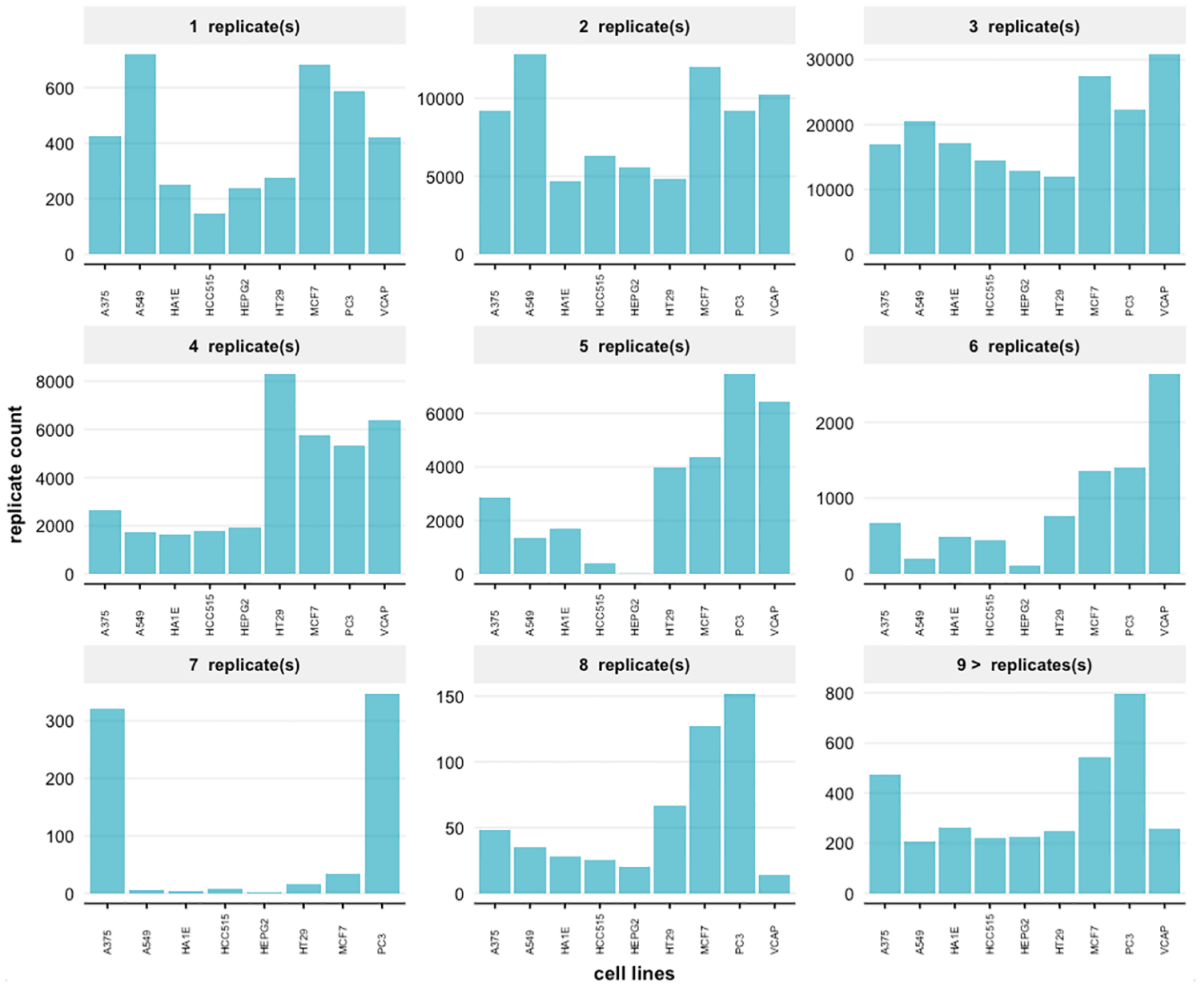
Distributions of experimental replicates for the signature profiles. The number of available replicates is shown for small molecule treatments in the LINCS L1000 data for 9 highly profiled cell lines.

Experimental replicates have been investigated and found to be useful in simulation and in boosting analysis [30] and decreasing the number of replicates will adversely affect the power of experiments [30, 31]. For this reason we studied the distribution of replicate experiments of the LINCS L1000 data. From this we find that the plate variation is ranging mostly between 1 to 8 replicates with the majority of samples having 3 replicates. There are also conditions for which more than 9 replicates have been generated, however, these are rare covering only 1% of all profiles, whereas 1 to 8 replicates cover 99%. The largest number of replicates observed is 27, e.g., found for cell line VCAP, drug Vorinostat, a dosage of 10um and a time duration of 24h. In Fig 3 we show the number of replicated experiments cross the 9 selected cell lines. The figure includes also information about 9 or more replicates and shows that the availability various greatly between the cell lines.

**Fig 4:**
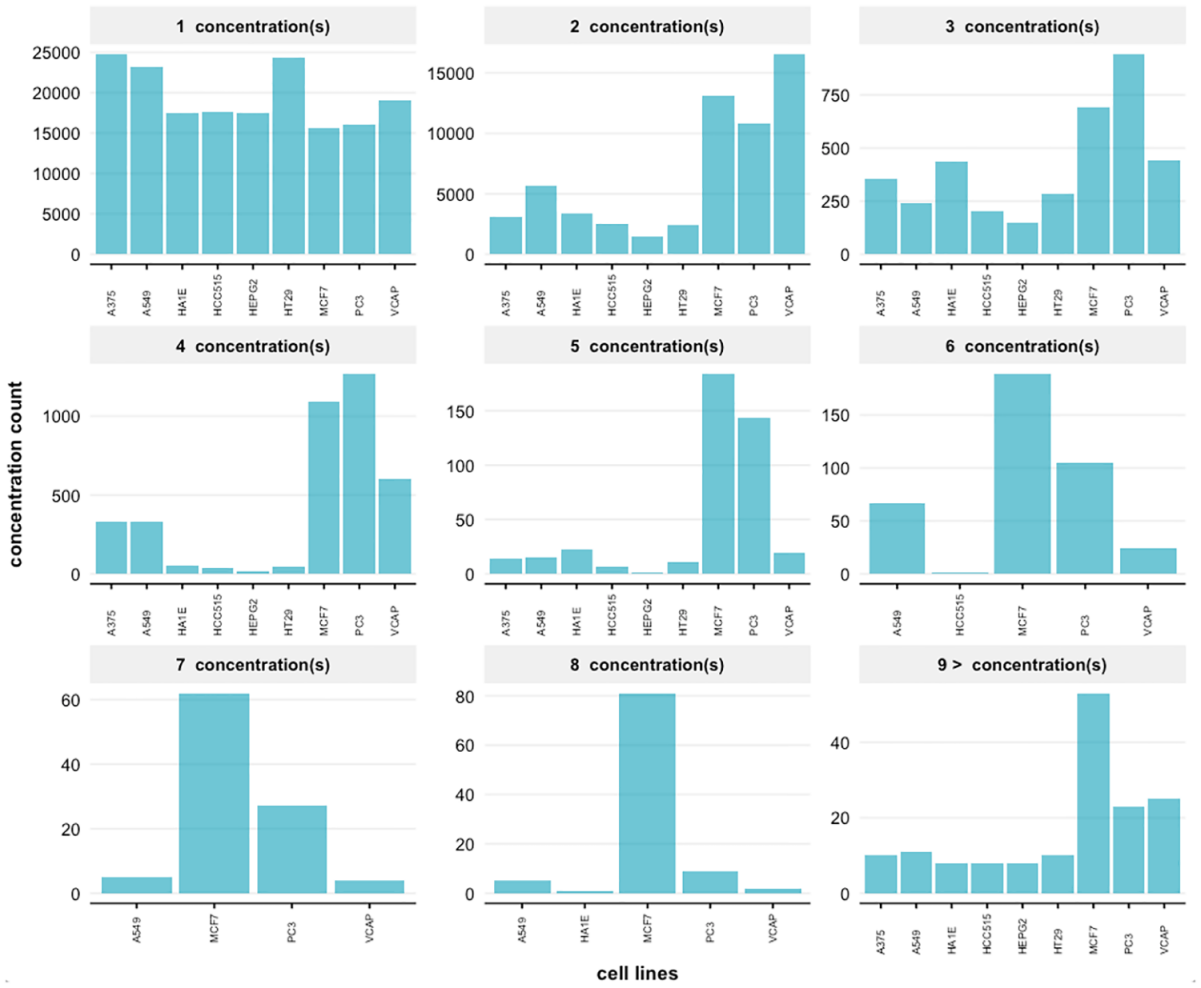
Distributions of unique dosages for the signature profiles. The number of available profiles is shown for different dosages (concentrations) of small molecules for 9 highly profiled cell lines.

Next, we show in Fig 4 results for the number of different dosages (concentrations) applied to the 9 highly profiled cell lines. The figure shows distributions for 8 different concentrations and 9 or more concentrations. However, almost 99% of the treated samples are measured for 1 to 8 different concentrations. From the available 49,400 perturbations, most of them were tested for a duration of 6, 24, 48, 96 and 120 hours. Overall, the number of cell lines per compound represented in the treatments ranged from 1 to 8 different time duration points (see Fig A in S1 File). Around 99% of the perturbations affected at least one gene significantly in a single cell line after treatment with the varying number of time points.

### B. Differentially expression of genes

#### Differentially expression of genes and small molecule diversity

Our next analysis focuses on the activity level of the gene expression data as quantified by differentially expressed genes. For this analysis we utilized the L1000 raw z-scores from the GEO repository and pre-processed these by using the R L1000 tools [32]. We utilized the signature meta-information in Signature Service API for selecting the same subset of 9 cell lines as in Table 2 (with highest signature counts across all cell lines). Here a signature for a small molecule is defined as a vector of z-score values, each representing differential expression of genes profile between small molecule treated samples and control samples. In total there are 169,239 z-score signature profiles for the 9 cell lines that satisfied the well- and plate-based quality control. This signature profile subset comprises 20,009 small molecules (out of 49,400 perturbations) that were repeatedly measured between 1 to 8 times. To further simplify the data and the quality of the analysis, we selected 6, 24 and 48*h* time points. In total this leaves us 158,054 signature profiles (i.e., any combination of the small molecule, time, and cell line) for our analysis. These signature profiles come from experiments that were carried out on 391 multi-wells, where 362 wells were used for treatment and 29 DSMO wells were for control vehicles.

In order to obtain the number of differentially expressed genes between treatment and control samples for each of the 384 plates we used the z-score signature vectors obtained from the Signature Service setting the z-score threshold to > 2.0 and < -2.0 for up- and down-regulated genes respectively. For measuring the signature type effects that have been shown to be robust in biological interpretations, we use the assigned z-score thresholds to measure the biological effects encoded in the gene expression data. We found that 19,957 small molecules from 20,009 that are used in 158,054 signature profiles yielded at least one gene that is significantly differentially expressed when compared with the corresponding control samples. We further found that 15,714 small molecules reveal significant differences for at least 50 genes, and 8, 211 small molecules are differentially expressed for at least 100 or more genes. Table 3 summarizes these results.

**Table 3:** Summary of z-score signature profiles resulting in differentially expressed genes (DEG) between treatment and control samples for the 9 cell lines in Table 2.

Differentially expressed genes	Signature profiles	Small molecules
No significant gene	24	19
At least 1 significant gene	158,030	19,957
At least 50 significant genes	58,739	15,714
At least 100 significant genes	23,867	8,211
**Total**	**158,054**	**20,009**

#### Cell type specific differentially gene expression

**Fig 5:**
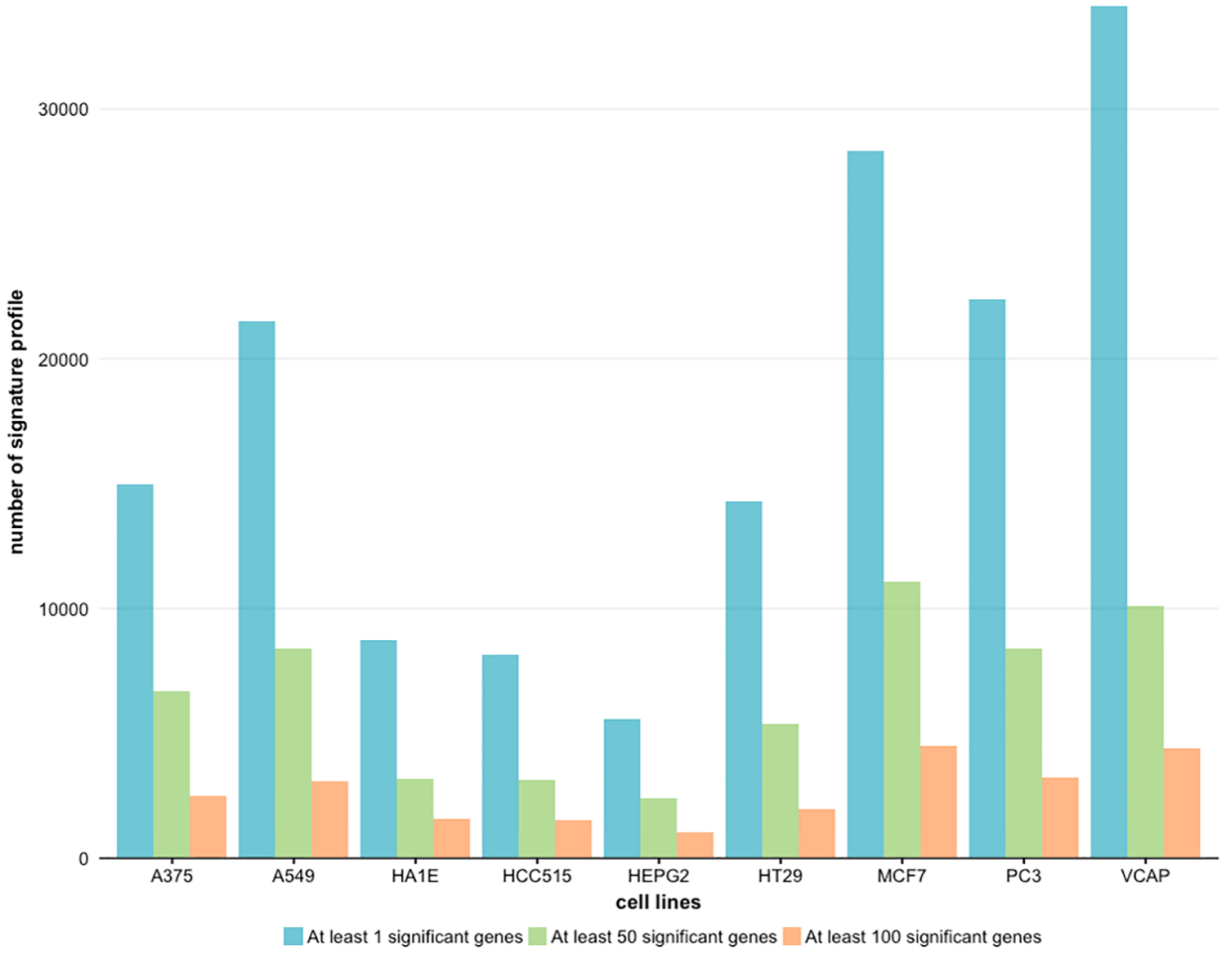
Number of significant profiles found when comparing signature profiles of treatment and control samples. The cell lines are categorized according to the number of DEGs and the DEG have been estimated based on the z-score signatures profiles.

Since not all cell lines measure the transcription effects of small molecules for the same time points, we subset the treatments according to cell lines and evaluate the number of significant genes for the 9 cell lines separately. In Fig 5 we show our results giving the number of signature profiles for each cell line for three categories. The three categories correspond to (I) at least one significant gene, (II) at least 50 significant genes, and (III) at least 100 significant genes when compared with vehicle controls. Since there were only 24 profiles with no significant genes in total, this category is not shown in the figure.

#### Dosage specific differentially gene expression

**Fig 6:**
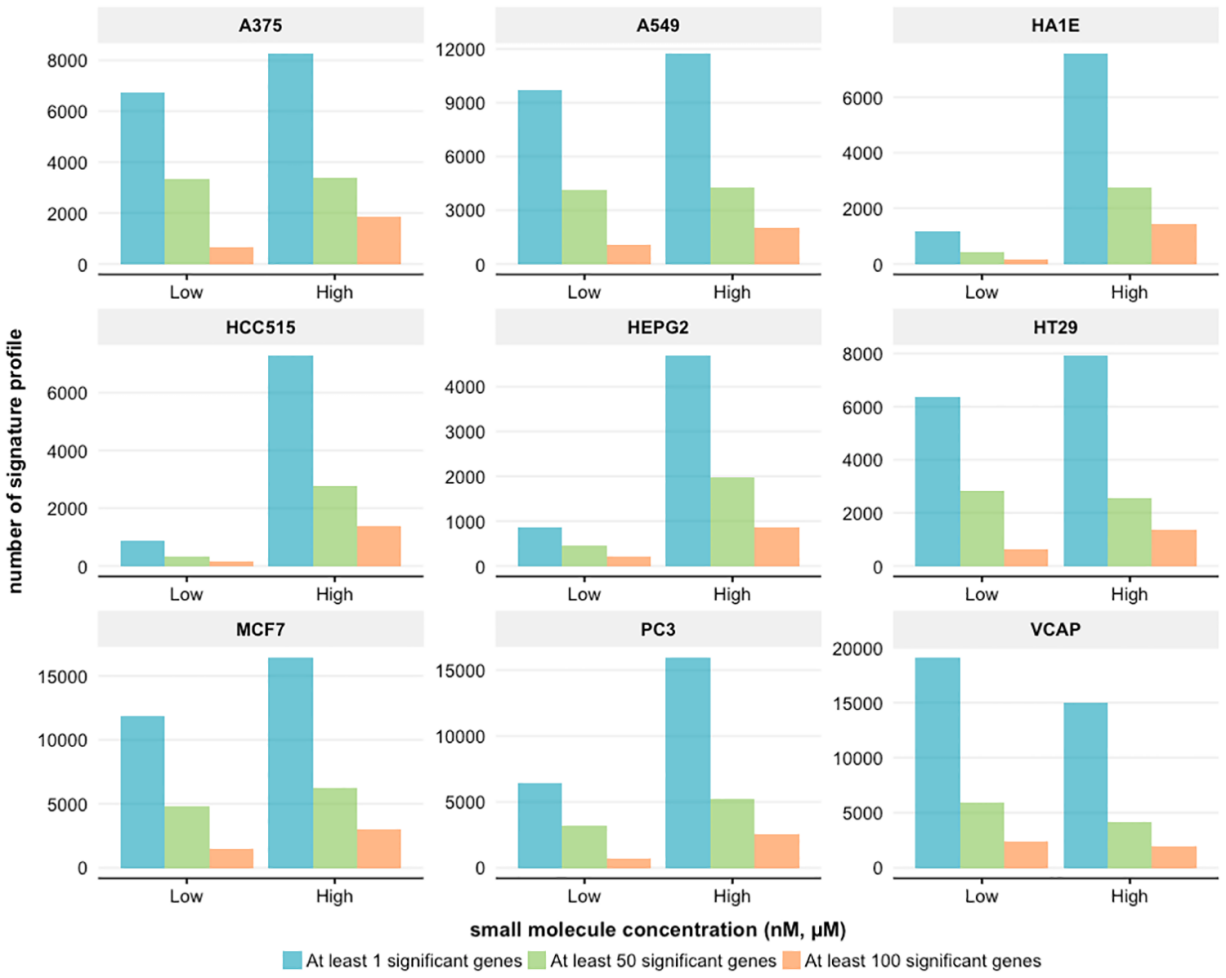
Dosage specific differentially gene expression. The differential expression of genes for 9 cell lines is shown categorized in Low and High dosages of small molecules.

For studying the effect of drug dosages we repeated a similar analysis as above. Specifically, we systematically classified the small molecule dosages into two categories for ‘low’ and ‘high’ concentrations. The ‘low’ concentration group contains all measurements in nanomolar (nM) and doses less than or equal to 5 micromolar (*μ*M) while the ‘high’ concentration group includes all measurements greater than 5 *μ*M. In total, we find 63,113 and 94,941 signature profiles for low and high dosages respectively. In Fig 6, the number of differentially expressed genes is shown for the 9 cell lines and the two dosage categories. From this we observe two different behaviors. First, the number of differentially expressed genes increases with time, e.g., cell line A375 or A549. Second, the number of differentially expressed genes decreases with time. This behavior is only observed for cell line VCAP. The first type of behavior is expected because higher dosages of drugs should result in more severe changes in the expression of genes. The reverse of this effect for cell line VCAP, a prostate cancer cell line, averaged over all drugs is counter intuitive and points to follow-up investigations.

#### Drug perturbation specific differentially gene expression

**Fig 7:**
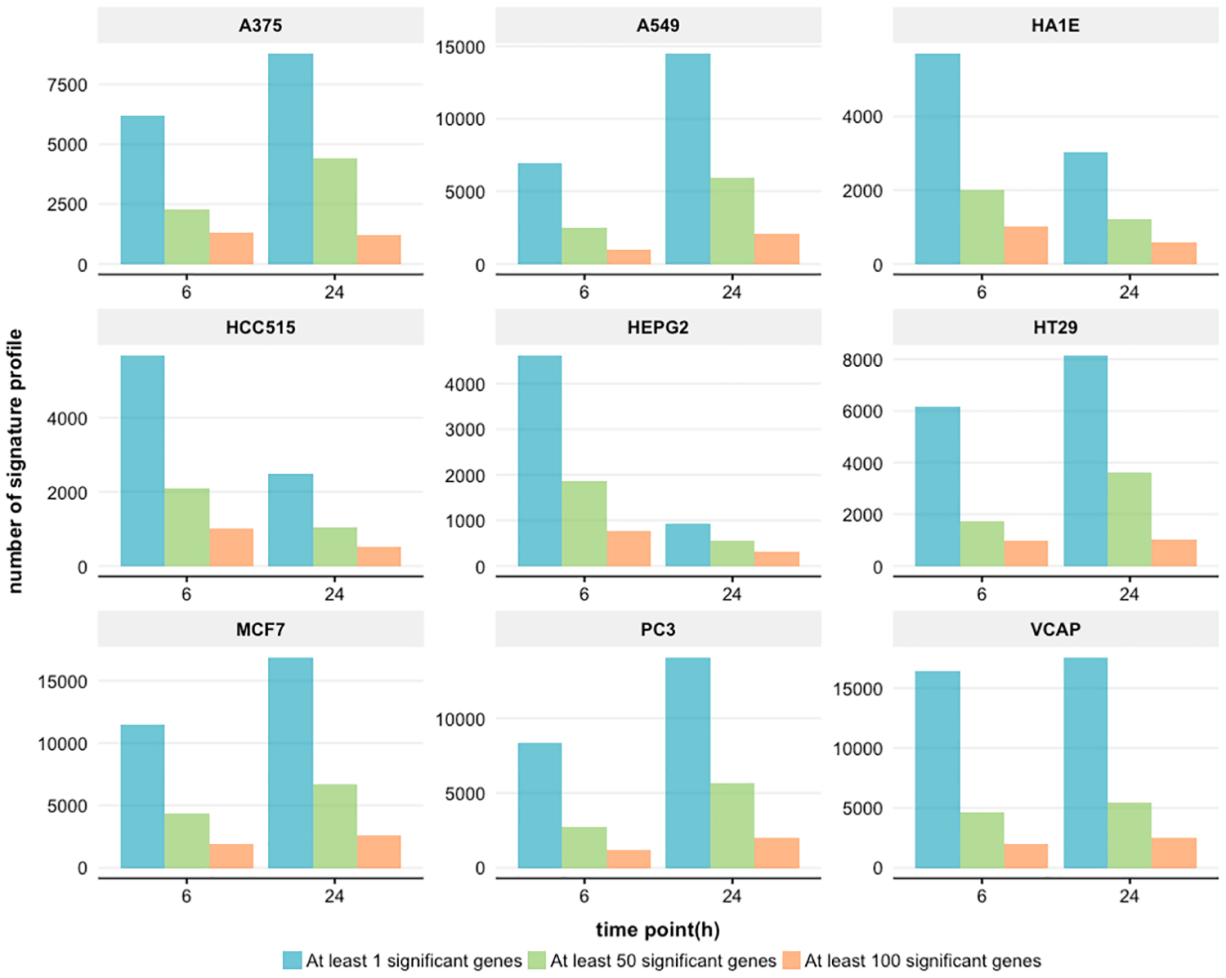
Drug perturbation specific differentially gene expression. The differential expression of genes for 9 cell lines is shown categorized in the time durations (6 and 24h) of drug perturbations.

Next, we analyze the number of differentially expressed genes according to the time duration of the treatment with small molecules. In Fig 7 we show results for 6 and 24 hours. From this we again observe two different behaviors. First, the number of differentially expressed genes increases with time, e.g., cell line A375 or A549. Second, the number of differentially expressed genes decreases with time, e.g., cell line HA1E or HCC515.

#### Changes in biological activity

**Fig 8:**
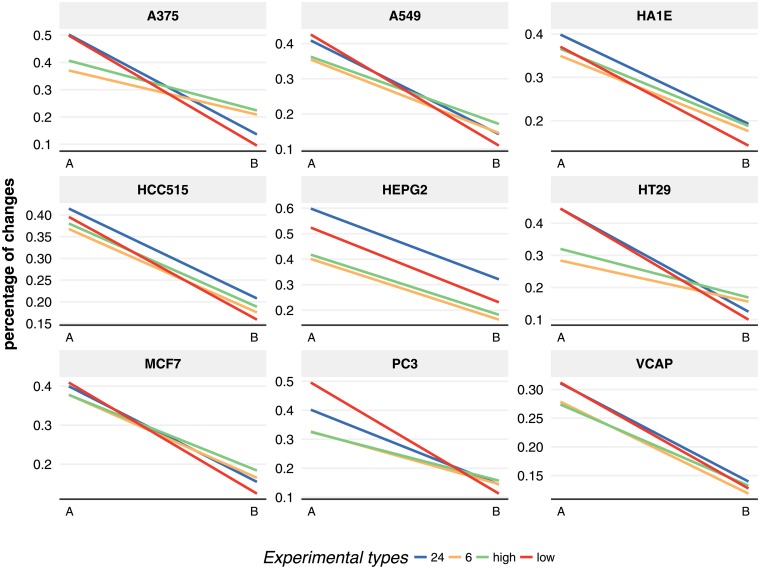
Changes of biological activity. Percentage changes in the number of significant profiles for the cell lines in dependence on the dosages and time points obtained from Figs 6 and 7. A. corresponds either to Low dosage or 6 hours and B. corresponds either to High dosage and 24 hours.

Finally, we compare the findings shown in Figs 6 and 7 to reveal changes in the biological activity of the corresponding cell lines. In order to do this, we estimate the fraction of change for each of the two categories ‘at least 50 significant genes’ and ‘at least 100 significant genes’ with respect to the category ‘at least 1 significant gene’. That means we are estimating

$$f_{A}^{50}=\frac{\#\text{profiles}(\text{at}\;\text{least}\;\text{50}\;\text{significant}\;\text{genes}|A)}{\#\text{profiles}(\text{at}\;\text{least}\;\text{1}\;\text{significant}\;\text{gene}|A)}$$ (1)

$$f_{A}^{100}=\frac{\#\text{profiles}(\text{at}\;\text{least}\;\text{100}\;\text{significant}\;\text{genes}|A)}{\#\text{profiles}(\text{at}\;\text{least}\;\text{1}\;\text{significant}\;\text{gene}|A)}$$ (2)

wheres A corresponds either to Low dosage or 6 hours and

$$f_{B}^{50}=\frac{\#\text{profiles}(\text{at}\;\text{least}\;\text{50}\;\text{significant}\;\text{genes}|B)}{\#\text{profiles}(\text{at}\;\text{least}\;\text{1}\;\text{significant}\;\text{gene}|A)}$$ (3)

$$f_{B}^{100}=\frac{\#\text{profiles}(\text{at}\;\text{least}\;\text{100}\;\text{significant}\;\text{genes}|B)}{\#\text{profiles}(\text{at}\;\text{least}\;\text{1}\;\text{significant}\;\text{gene}|A)}$$ (4)

whereas B corresponds either to high dosage and 24 hours. This results in 8 percentage values for each cell line, 4 values from Fig 6 ($f_{\text{Low}}^{50}$, $f_{\text{Low}}^{100}$, $f_{\text{High}}^{50}$, $f_{\text{High}}^{100}$) and 4 values from Fig 7 ($f_{\text{6}\;\text{hours}}^{50}$, $f_{\text{6}\;\text{hours}}^{100}$, $f_{\text{24}\;\text{hours}}^{50}$, $f_{\text{24}\;\text{hours}}^{100}$). From these we obtain four straight lines per cell line defined by the pairs ($f_{\text{Low}}^{50}$, $f_{\text{High}}^{50}$) (green line in Fig 8) and ($f_{\text{Low}}^{100}$, $f_{\text{High}}^{100}$) (blue line in Fig 8) for dosages and ($f_{\text{6}\;\text{hours}}^{50}$, $f_{\text{24}\;\text{hours}}^{50}$) (red line in Fig 8) and ($f_{\text{6}\;\text{hours}}^{100}$, $f_{\text{24}\;\text{hours}}^{100}$) (orange line in Fig 8) for time points. Overall this means Fig 8 shows a summary of the fraction (percentage) of changes in the biological activity in dependence on different experimental conditions.

From Fig 8 we obtain two major observations. First, regarding the slope of the four straight lines, we observe that either these are parallel or they intersect each other. A parallel behavior is observed for cell line HEPG2 or VCAP, whereas an intersection is observed for HT29 or A375. This means that changes in the drug dosages has a nonlinear effect for cell line HT29 or A375 compared to, e.g., cell line HEPG2 or VCAP, if contrasted with changes in the time points. The second major observation from Fig 8 is the change of the top y-scale. For instance for cell line HEPG2 we find the highest percentage change of 60% for 24 hours, whereas for cell line VCAP this is only slightly over 30%. The difference is almost a factor of two in the activity changes.

## Discussion

In this study, we analyzed the LINCS L1000 dataset by characterizing different experimental variables including cell types, time points, and dosages. We performed our analysis for two different layers. In layer one we focused on distributional characteristics of signature profiles whereas in layer two we focused on biological activity changes as measured by the number of differentially expressed genes.

Despite the fact that the LINCS L1000 dataset contains information for 71 cell lines, the vast majority of data is available for 9 cell lines only, namely A375, A549, HCC515, HA1E, HEPG2, HT29, MCF7, PC3 and VCAP, as can be seen from Fig 2 and Table 2. Each of these cell lines contains more than 20,000 signature profiles which enables excellent analysis opportunities. In contrast, for 46 cell lines less than 500 signature profiles are available. This means the utility of these 46 cell lines for any pharmacogenomic application is severely limited. Overall this means, that only 12% of all cell lines enable comprehensive large-scale data-driven pharmacogenomic applications.

For the number of replicates, we found that 2, 3 and 4 replicates are the majority for the 9 highly profiled cell lines, see Fig 3. However, also the number of replicates vary greatly between the cell lines. For instance, for HT29 there are over 8000 profiles with four replicates available whereas for A549 there are less than 2000 profiles, which means the difference is a factor of four. For studies requiring a very large number of replicates the cell lines MCF7 and PC3 are preferable because these cell lines provide experimental condition with over 9 replicates. To a lesser extend this is also true for A375. This information is important for planning an analysis in order to prevent an underpowered analysis [33] and ensure accurate estimations in a downstream analysis [34].

From the distribution of dosages (concentrations of drugs or small molecules) we found that most of these are used only with one or two concentrations, see Fig 4. However, for cell line MCF7 small molecules have been applied for even more than 9 different concentrations. Overall, the screening character of the LINCS project is well reflected by the distributions for different concentrations across the 9 cell lines in Fig 4 because of the high variability in the resulting number of signature profiles.

The second part of our analysis focused on the differentially expression of genes. As an overall results we find 24 profiles without any significant gene, 158,030 profiles with at least 1 significant gene, 58,739 with at least 50 significant gene and 23,867 profiles with at least 100 significant genes, see Table 3. For these numbers we averaged over all cell lines and experimental conditions. From this analysis we can conclude that 99.99% of all signature profiles contain at least some activity changes induced by the applied perturbations. Interestingly, the induced activity changes in the expression of genes seem to be moderate because 62% of all signature profiles contain between 1 and 49 significant genes.

It has been pointed out by Iorio et al. [35] that a compound can show inconsistent transcriptional effects when applied across different cell lines, its biological effect may be differentiated when merging gene expression values from different cell lines. Therefore, the compounds that were used to assess the effect on the cell lines may hold a bias towards a particular biological effect, since a cell line might react differently to certain treatment [36–38].

By zooming into the individual cell lines, see Fig 5, these overall observations are confirmed, although, there are certainly noticeable variations in the level of activity changes. For instance, for cell line A375 we find a decrease of around 40% from the number of significant profiles in category one to category two, whereas for cell line VCAP this decrease is only about 30%. This is actually a desirable observation because it means the LINCS data reflect that natural variability and sensitivity of the different human cell lines.

Next, we performed a detailed analysis studying the influence of the dosage and the time points on the individual cell lines. For the dosages we observed two different behaviors, see Fig 6. Behavior one corresponds to an increase in the number of significant profiles when going from low to high dosages, across the three gene categories, e.g., for cell line A375 or HA1E. In contrast, behavior two corresponds to a decrease. Interestingly, this behavior is only observed for cell line VCAP.

For the time points we obtain similar results, see Fig 7. For the first behavior the number of differentially expressed genes increases with time, e.g., cell line A375 or A549. For the second behavior the number of differentially expressed genes decreases with time, e.g., cell line HA1E or HCC515.

An explanation for this is that either lower or higher concentration treatments do not kill cells rapidly. Due to this reason, they should be tested for a longer period of time/days. In experimental setup of the L1000 data it is possible that a higher concentration might not killed the entire population rather induced a resistance population in which cell cycle is not be arrested. Furthermore, it should be also noted that PC3 (high metastasis) and VCAP (moderate) are not in the same state.

Finally, we compared the influence of dosage changes (Fig 6) with the influence of time point changes (Fig 7) in order to reveal changes in the biological activity of the corresponding cell lines and summarized these findings in Fig 8. From this we obtained two major observations. First, either the slope of the four experimental types occurs in parallel or they intersect with each other. Second, the y-scale is not the same for all cell lines. These results demonstrate the nonlinearity of the biological activity of the cell lines as a function of the different experimental conditions (types) and, hence, show the biological complexity of the transcription regulation.

All these results allow to gain insights that go beyond the mere features of gene expression data, e.g., providing information about the number of samples or number of drugs used for perturbing the cell lines. Instead, the second part of our study provides information for selecting cell lines with respect to their activity profiles. This information is important for the design of any pharmacogenomic study regardless of their particular goals because it is the biological activity of genes that decides about the effect of drugs.

Interestingly, in a previous study it has been shown that using additional cell lines provides more information about the compound-induced biological effects when different time points are used in the experimental design [39]. We found two time points (6h and 24h) yielded the most number of significant genes (see Fig 7) in the L1000 data. Therefore, the time point coverage can provide an understanding of how the L1000 data is represented at the gene level. Moreover, the combination of MCF7, VCAP, A549, HT29, and PC3 cell lines covers the majority of the transcriptional effects.

Overall, the LINCS L1000 data provides a rich and valuable source of compound-induced data that addresses some of the problems as mentioned for the CMap data [14, 16]. For example, a limited number of replicates, batch effect sizes and small number of profiles, now are all increased and improved in the L1000 assay. However, there are still shortcomings: First, most of the compounds are profiled at a high single dose only, causing different variability in dosage measurements. Second, the dataset does not explicitly follow the conventional settings of using experimental variables which are needed in a genome-wide transcriptional profiling study [40], but measure only 978 gene transcripts while the rest of the transcriptome was estimated by a model. Finally, the compounds are neither from primary-screening libraries such as FDA-approved nor the molecularly targeted and not highly selective agents that would be of particular interest for researchers [41].

## Conclusion

In this paper, we used the Big Data from the LINCS project to explore different experimental settings, such as cell line coverage, time points and dosages using a data pipeline to assess compound-induced transcriptional effects. As a result, first, we provided summary statistics for distributional characteristics of gene expression signature profiles from all cell lines and their perturbagents. Second, we revealed changes in the differential expression of genes manifesting the biological complexity of the perturbagents. As a result, our analysis hopefully helps in harnessing the overwhelming complexity of the LINCS data providing guidance for the experimental design of follow-up studies, e.g., by selecting appropriate cell lines.

Given the limitations of previous datasets such as the CMap [14, 16], our analysis suggests that the L1000 data provide a good opportunity for the characterization of the compound-induced transcriptional effects. Given the volume and complexity of this dataset for drug discovery, it is necessary to understand the potential of the L1000 dataset and how it can be used in a drug research setting where every step is driven by data and rigorous data models. For example, the selection of appropriate tools to access, analyze and create models using the dataset to validate hypotheses. More efficient ways are expected to quickly transform Big Data discoveries into clinical applications.

## Supporting information

S1 File This file is provided as a zip file containing figures and R code related to the manuscript. (ZIP)
