# Supplementary material for: Harnessing the biological complexity of Big Data from LINCS gene expression signatures
Source: PLoS One. 2018 Aug 29;13(8):e0201937. doi: 10.1371/journal.pone.0201937 (PMC6114505; doi:10.1371/journal.pone.0201937)
Supplement: S1 File — (ZIP) [file pone.0201937.s001.zip › S1-file.pdf]

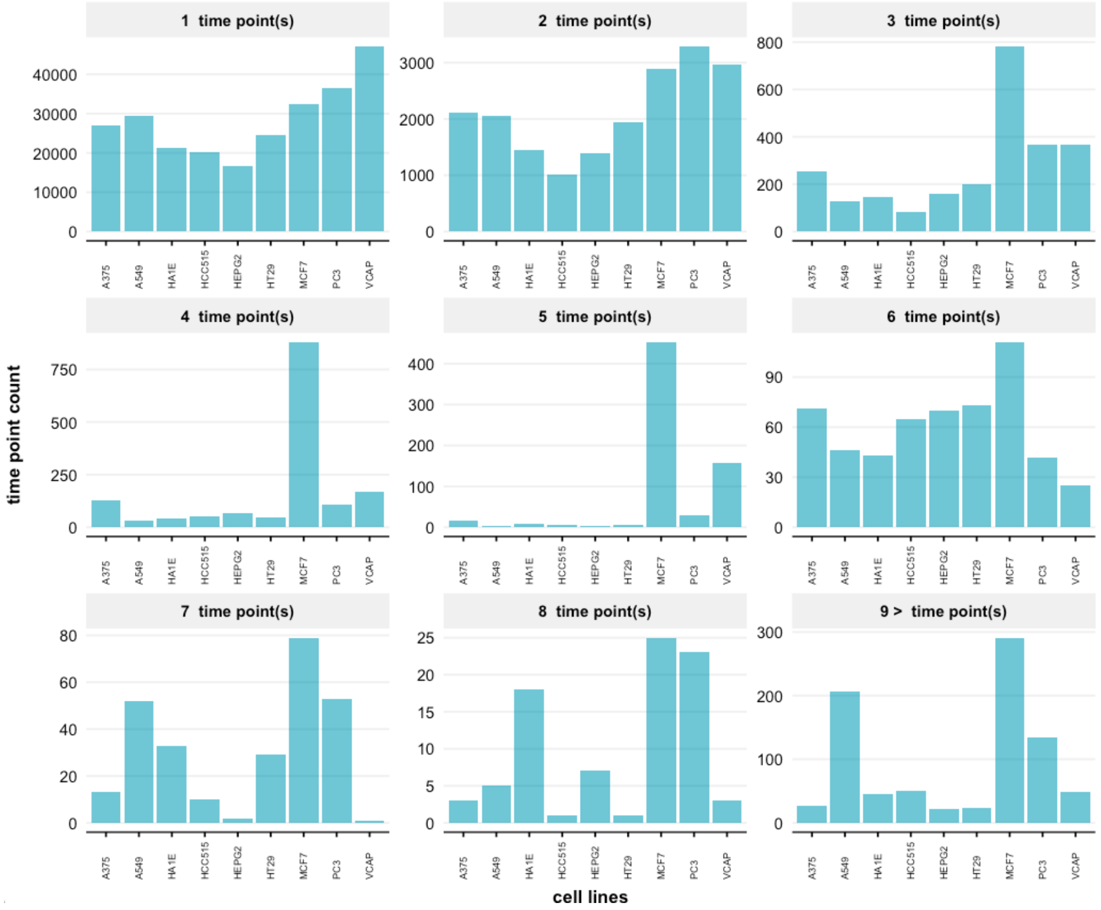

Figure 1: Number of different time points measured on the 9 cell line subset. The number of cell lines per compound represented in the treatments ranged from 1 to 8 different time points count out of 14. Around 99% of the perturbagens affected at least one gene significantly in a single cell line after treatment with the different number of time points.
